# Desert Ants Learn to Avoid Pitfall Traps While Foraging

**DOI:** 10.3390/biology11060897

**Published:** 2022-06-10

**Authors:** Adi Bar, Chen Marom, Nikol Zorin, Tomer Gilad, Aziz Subach, Susanne Foitzik, Inon Scharf

**Affiliations:** 1 School of Zoology, The George S. Wise Faculty of Life Sciences, Tel Aviv University, Tel Aviv 6997801, Israel; adibar1@mail.tau.ac.il (A.B.); chenmarom@mail.tau.ac.il (C.M.); nikolzorin@mail.tau.ac.il (N.Z.); tomergilad@mail.tau.ac.il (T.G.); azizsubach1@gmail.com (A.S.); 2 Institute of Organismic and Molecular Evolution, Johannes Gutenberg University Mainz, 55128 Mainz, Germany; foitzik@uni-mainz.de

**Keywords:** antlions, desert ants, movement, pitfall traps, spatial learning, shadow competition

## Abstract

**Simple Summary:**

Animals living in nests leave their nests to search for food and often use constant routes. We tested how workers of ant colonies cope with pitfall traps placed on their way to food. Such pits can represent those dug by the ant-hunting pit-building antlions. The pitfall traps delayed the arrival at the food and increased the workers’ tracks, but the ants improved in searching after accumulating experience. Furthermore, workers learned to avoid falling into the pits with experience. Removing or adding pits led to a fast change in the worker behavior and they ignored the past conditions, except for tracks that were longer than expected, after pitfall traps were removed. The ants fell much more frequently into pits closer to the arena entry, suggesting that such positions are especially profitable for sit-and-wait predators, ambushing such ants.

**Abstract:**

Central-place foragers, such as social insects or nesting birds, repeatedly use the same routes from and to their nests when foraging for food. Such species forage more efficiently after accumulating experience. We examined, here, a relatively neglected aspect of such an improvement with experience—the avoidance of pitfall traps. Similar pits are built by antlions, which co-occur with the ants, but they also resemble other natural obstacles. We used the desert ant *Cataglyphis niger*, common in sandy habitats, and allowed it to forage for three successive runs for a food reward. Ant workers discovered food more slowly and in smaller numbers when pits were in their path. Pit presence also led to longer tracks by ants and slower movement. However, with experience, the ants fell into such pits less often and reached the food more quickly. To understand how past conditions affect current behavior, we investigated whether removing or adding pits led to a different result to that with a constant number of pits. Workers adjusted their behavior immediately when conditions changed. The only carryover effect was the longer tracks crossed by workers after pit removal, possibly resulting from the mismatch between the past and current conditions. Finally, the workers were more likely to fall into pits that were closer to the nest than those that were further away. This is a good example of the advantage that ambush predators can derive from ambushing their prey in specific locations.

## 1. Introduction

Foraging is a vital behavior for both survival and reproduction. Foraging gains mostly depend on the food’s caloric value and the forager’s nutritional requirements [1,2,3]. The costs are more diverse and include the energy and/or time spent, the missed-opportunity cost, and the predation risk [4,5,6]. The latter cost can be expanded to refer also to other misfortunes that can happen, leading, for example, to injury or getting lost [7,8,9].

Central-place foragers, such as nesting birds, rodents living in burrows, and social insects, leave a nest/burrow to search for food [10]. Inevitably, they retake the same routes, especially close to their nest. Such fixed routes are even more evident if they repeat traveling to the same food patches when those are renewable or slowly depleted [11,12,13]. Predators can take advantage of such routes and ambush prey where the probability of encounters is relatively high. Sit-and-wait predators, such as snakes or spiders, choose ambush places where they expect to encounter prey more frequently [14,15]. While the predators aim at a spatial overlap with their prey, the prey should do its best to avoid it [16]. Central-place foragers do not only learn to avoid predators but also become more efficient at finding their way to food patches and back to the nest [17]. This contributes to saving time, allows faster exploitation of food, and reduces exposure to different sorts of danger. Thus, spatial orientation and learning are important foraging components of many central-place foragers (e.g., [18,19,20]).

Ants are a fine example of central-place foragers. When foraging, ant workers are exposed to numerous risks, such as predation, parasite infestation, injury, dehydration, or getting lost [8,14,21,22,23]. Spiders specializing in ant predation ambush ants on the routes to food [24]. Pit-building antlions and wormlions dig pitfall traps in loose soil, such as sand or loess, and hunt ant workers too [25,26]. The capture of an ant worker falling into such pits is not certain and it depends on several factors, such as the soil type and humidity level, pit size and shape, the predator actions, and the ant worker size [27,28,29,30]. The capture likelihood of some ant species is further reduced owing to rescuing behavior by their nestmates, probably attracted to a volatile pheromone emitted by the trapped worker [31,32]. When ant workers are recruited, the predator might find itself becoming the prey [33]. In any case, since capture success is not certain, i.e., ant workers falling into pitfall traps and managing to get out, they may learn to avoid such traps.

A rather neglected question is the response of foragers to changing conditions during foraging or the importance of changing direction. For example, plants grow more roots in soils when conditions improve than when they remain stable [34]. Not only the direction of change but also the rate of change may be relevant. Antlions, for example, relocate their pits when prey abruptly stops arriving, whereas a gradual decrease does not induce relocation [35]. Generally, foragers experience changing conditions, and, to better understand their behavior, it is recommended to examine how they respond to such changes.

We examined the effect of pitfall traps on foraging for food in the desert ant *Cataglyphis niger* and whether the response to such traps changes with experience. A similar question has been studied in the congeneric species *C. iberica*, which changes foraging routes to avoid falling into pits [36]. Desert ants forage individually (no pheromone-based recruitment) for scattered food and use an array of tools, such as a vector pointing in some direction combined with distance estimation (“odometer”), a panoramic view of the environment, and path integration for navigation to the food source and back to the nest [37,38,39,40,41]. Many such species occur in sandy habitats, in which pits dug by pit-building predators as well as other opportunities to get trapped exist [42,43,44]. The congeneric species *Cataglyphis cursor* or *C. piliscapa* rescue workers when trapped [42,45].

As falling into the pitfall traps is often not lethal, we expected that ant workers would learn to avoid pits with experience and would forage more efficiently. In addition, we examined the effect of improving or deteriorating conditions (removing or adding pitfall traps) on the ant worker performance. We expected the deteriorating conditions to make more workers fall into pits and to reach the food reward more slowly than the control because the workers were first trained that a straight path leads to the food reward, a path that is of no use after adding pits. The studied species had already demonstrated its ability to improve quickly in maze-solving owing to a mixture of spatial learning and elevated searching motivation [46,47,48].

## 2. Materials and Methods

### 2.1. Collection and Maintenance of Ant Colonies

We collected eight colonies of *C. niger* for each of the two experiments conducted. The colonies were collected from Tel Baruch sand dunes, an enclave of a disturbed natural area in the northwestern corner of Tel Aviv (32.132° N, 34.788° E). See [49] for the vegetation cover and other ant species in this habitat. Antlions are also present in these sand dunes [50]. The colonies were split a week after collection into either three groups in the first experiment or two groups in the second experiment, each group comprising 40 workers and neither a queen nor a brood. They were kept in acclimation boxes (18.5 × 18.5 × 10 cm) with access to water in tubes for a week under 14:10 L:D photoperiod at ~28 °C.

### 2.2. Experimental Design

**Figure 1:**
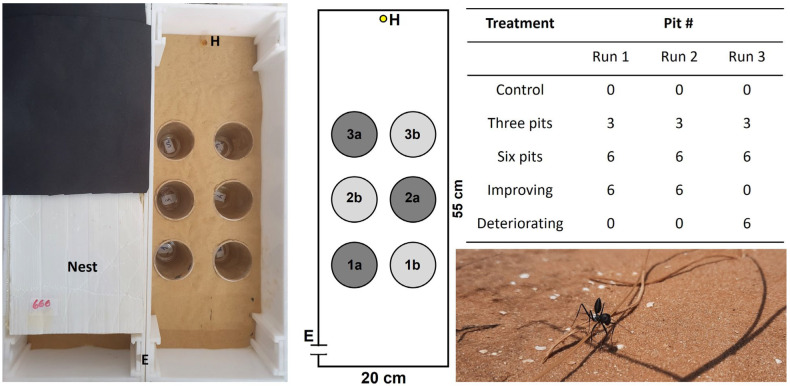
Left: A photo and a scheme of the test arena. The two horizontally parallel lines (E) stand for the entry to the arena from the nest. The small yellow circle (H) stands for the food reward (0.5 g honey). The large grey circles (1a,b, 2a,b, and 3a,b) stand for the six pits. In the three-pit treatment only 1a, 2a, and 3a were deployed in a zigzag arrangement, and the other pits were covered with sand. Right: The five treatments applied in the two experiments and the number (#) of pits in each of the three experimental runs. Lower right corner: A *C. niger* worker in its natural habitat.

The acclimation box was connected to an arena (55 × 20 cm; Figure 1) with a door (a hole of 1 cm in diameter that could be closed) separating the two. The arena was filled with a uniform layer of 1 cm deep sand collected from the natural habitat. On the arena’s far side, we placed a cut Eppendorf lid with 0.5 g honey. The arena contained no, three, or six pitfall traps (diameter of 7 cm, depth of 10 cm below the arena level), depending on the treatment (see below). The pits were placed below holes in the arena, so they were not observable from the ant worker’s perspective. Each group of workers was examined in three successive runs. Each run commenced by opening the door between the acclimation box and the corridor and between the corridor and the arena. Each run lasted either 50 min or 10 min after the arrival of the first ant worker at the food reward, the shorter of the two. Each run ended by burying the workers that fell into the pits with a few cm of sand in order to increase the negative feedback of falling, and after about two minutes they were placed back in the nest using featherweight forceps. Other workers in the arena were also brought back to the nest at this stage. We did not notice any adverse effect of gently picking up the workers with forceps and placing them in the nest. The interval between successive runs was 30 min, during which the sand in the arena was mixed and rearranged. In the first experiment, we applied three treatments: control (no pits), three pits, or six pits (Figure 1). The three pits were arranged in a ‘zigzag’ shape. The arena remained identical throughout the three successive runs. In the second experiment, we simulated either improving or deteriorating conditions. We deployed either six or no pits in the first and second run, after which the conditions were switched during the third run. During the experiment, we measured three response variables: food-discovery time (the time required for the first worker to reach the food reward measured from the experiment beginning), worker arrivals (the total number of workers arriving at the food reward), and workers falling (the total number of workers falling into all pits). All runs were filmed. Based on these films, we evaluated the track length of the first worker arriving at the food reward. To do so, we placed a transparent slide on the computer screen and documented, with a thin marker, the worker’s track from the maze entrance until its arrival at the food. Then, we used the software ImageJ [51] to measure the track length. We did so for the third run of the first experiment and the improving conditions treatment of the second experiment. We referred only to the third run for two reasons: more workers arrived at the food reward on the third run, and we were interested in the cumulative effect of the treatments applied. The experiment was conducted under light and ~28 °C.

### 2.3. Statistical Analysis

*First experiment: Constant numbers of pits:* We examined the effect of treatment (six pits, three pits, or no pits) and run (1 or 3) on food-discovery time, worker arrivals, and workers falling. Regarding the number of workers falling into pits, the control (no pits) was omitted from the analysis. Colony ID was referred to as a random variable. Food-discovery times were square-root transformed and this transformation led to test residuals that did not deviate from a normal distribution (Shapiro–Wilk test statistic: 0.976, *p* = 0.409). Worker arrivals were transformed using the 1/(1+worker arrivals) function, which led to test residuals that did not deviate from a normal distribution (Shapiro–Wilk test statistic: 0.964, *p* = 0.149). Workers falling were square-root transformed leading to no deviation of the residuals from a normal distribution (Shapiro–Wilk test statistic: 0.973, *p* = 0.328). Next, we focused on the third run and examined whether the track lengths and movement speed (i.e., track length divided by the time from entry to the maze until food discovery) of the first worker arriving at the food reward differed among treatments. We used two mixed linear models, with treatment (0, 3, or 6 pits) as a fixed factor, colony as a random factor, and either track length or movement speed as the response variable. Track lengths were log_10_-transformed, which led to no deviation of the residuals from a normal distribution (Shapiro–Wilk test statistic: 0.957, *p* = 0.536). Movement speed did not require a transformation (Shapiro–Wilk test statistic: 0.974, *p* = 0.876).

Finally, we tested whether workers fell more often into pits closer to the arena entrance (Figure 1). Pits were arranged at three distances from the arena entrance (distance to pit center): 15 cm, 25, and 35 cm (Figure 1). To this end, we summed the number of workers falling into the pits at each distance for the six-pit treatment and used the row number for the three-pit treatment, as each pit was located at another distance. We focused on the third run only and used a linear mixed model with pit position (1, 2, or 3) and treatment (three or six pits) as fixed factors, colony as a random factor, and the number of workers falling per pit distance (square root transformed) as the response variable.

*Second experiment: Changing numbers of pits:* The goal here was to examine the effect of change on food-discovery time and worker arrivals. To this end, we examined the effect of treatment (improving conditions, deteriorating conditions, and the control, taken from the first experiment) on the two above-mentioned response variables. As we were interested in the difference between the first and third run, when the conditions changed, we created two new variables: Δfood-discovery time = food discovery time in run 1 - food discovery time in run 3, and Δworker arrivals = worker arrivals in run 3 - worker arrivals in run 1. We used linear mixed models with colony as a random variable. The residuals of Δworker arrivals did not differ from a normal distribution (Shapiro–Wilk test statistic: 0.939, *p* = 0.152), but Δfood-discovery time was square-root transformed after adding the minimal value (to avoid negative values), leading to no deviation of the residuals from a normal distribution (Shapiro–Wilk test statistic: 0.956, *p* = 0.367).

To examine whether changing conditions differed from constant conditions, we conducted the following comparisons: (1) The food-discovery time and the number of workers falling into pits in the third run in the six-pit treatment vs. those in the deteriorating conditions treatment; (2) the food-discovery time, track length, and movement speed in the third run of the control (no pits) vs. those in the improving conditions (no pits as well). We did so by using separate Mann–Whitney tests, as each colony was represented only once in this case.

All statistical analyses were carried out using Systat v. 13 (Systat Software, San Jose, CA, USA). Multiple testing was accounted for using the Benjamini–Hochberg false discovery rate method (a false discovery rate of 10%; [52]). All significant results held despite the correction. The dataset appears in the Supplementary Materials (Excel File S1).

## 3. Results

### 3.1. First Experiment: Constant Numbers of Pits

**Figure 2:**
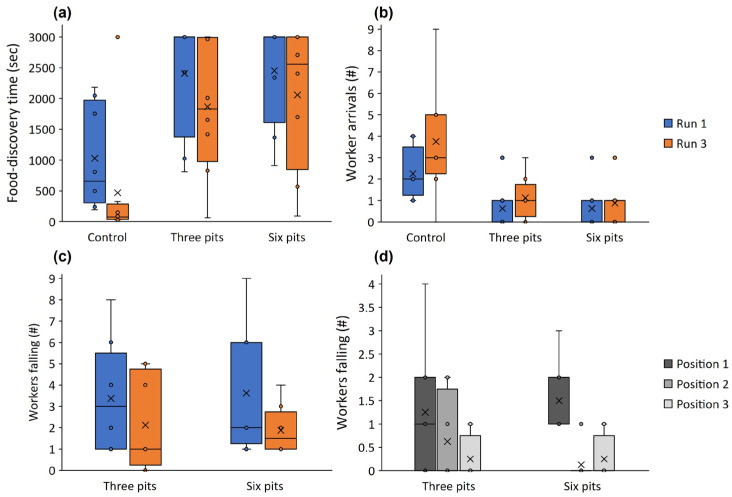
Food-discovery time (**a**) and the number of workers arriving at the food reward (**b**) in the first run (blue) and third run (orange) in the control, three-pit, and six-pit treatments. (**c**) The number of workers falling into pits in the three-pit and six-pit treatments in the first and third run (blue and orange, respectively). (**d**) The number of workers falling into pits in positions 1, 2, and 3 (closer and further away from the arena entry, respectively) when three pits are deployed in a zigzag pattern and when six pits are deployed. Lines and × signs in the box centers stand for the median and mean. Boxes, error bars, and dots stand for the quartiles, the range without outliers, and outliers.

**Figure 3:**
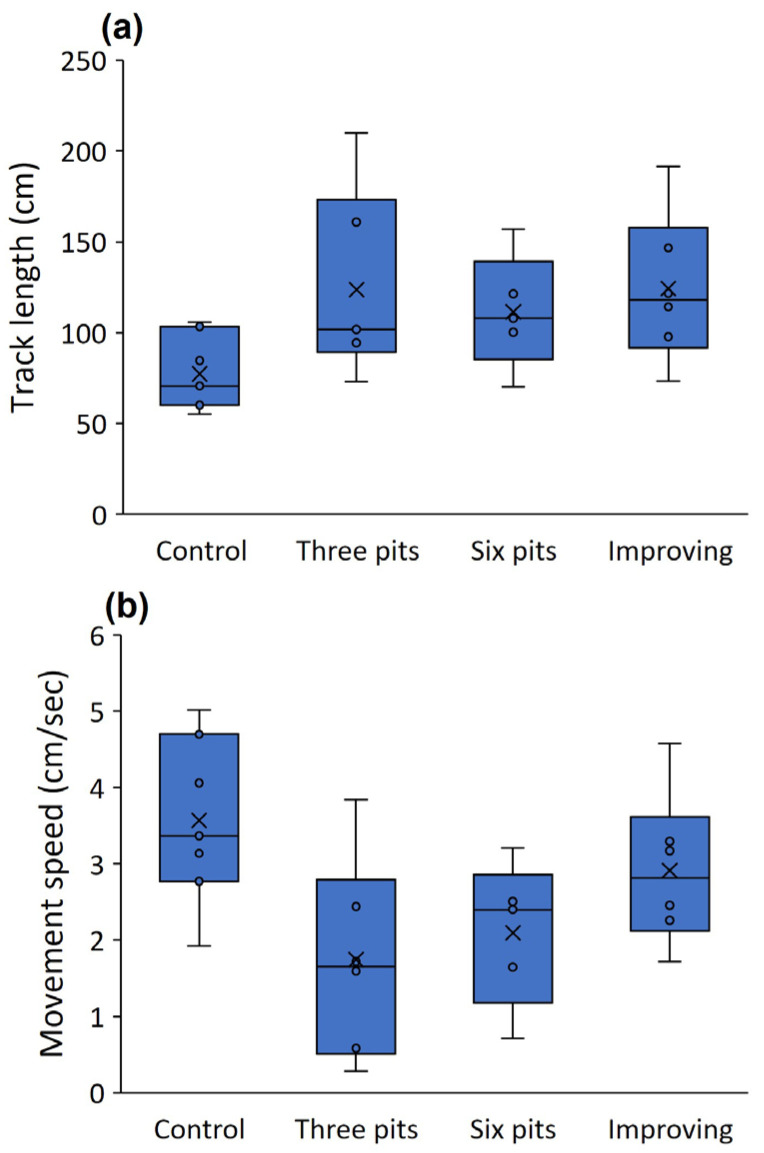
Track lengths (**a**) and movement speed (**b**) of the first worker arriving at the food reward in the three treatments of the first experiment (control, three pits, and six pits) and in the improving conditions (second experiment). Only the third run is presented. Lines and × signs in the box centers stand for the median and mean. Boxes and error bars stand for the quartiles and the range.

**Figure 4:**
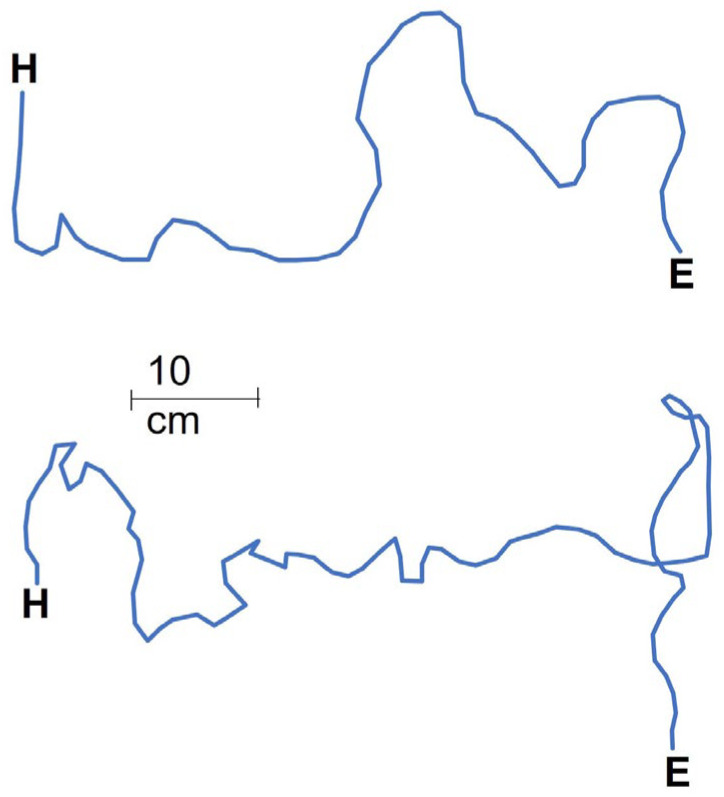
Two examples (above: control, no pits; below: six pits) of movement tracks from the arena entry (E) to the food reward (H) during the third run.

Workers discovered the food around three times faster in the control (no pits) than in either of the other treatments (three and six pits; *t* = 4.744 and 5.039, *p* < 0.001 for both; Figure 2a). The difference between the three- and six-pit treatments was minor. Food-discovery was about a third faster in the third run than the first run *(t* = −2.484, *p* = 0.018; Figure 2a). More workers (more than three times) arrived at the food reward in the control than in the two other treatments (three and six pits; *t* = 3.858 and 4.318, *p* < 0.001 for both, respectively; Figure 2b), but the run number had no effect on worker arrivals *(t* = −1.627, *p* = 0.112). The difference in the number of workers falling between the three- and six-pit treatments was not significant *(t* = −0.407, *p* = 0.688), but about 75% more workers fell into pits in the first run than in the third run *(t* = −2.065, *p* = 0.051; Figure 2c). Tracks were shorter in the control than the three-pit treatment by around 60% *(t* = 2.466, *p* = 0.039; Figure 3a) but the control did not differ from the six-pit treatment *(t* = 1.950, *p* = 0.087). See Figure 4 for examples of the two tracks. Similarly, in the control, movement speed was around double that in the three-pit treatment *(t* = −2.895, *p* = 0.020; Figure 3b) but did not differ from the six-pit treatment *(t* = −2.223, *p* = 0.057). Pit position had a strong effect on the number of workers falling: over three times more workers fell into pits in the first position than in the second or third positions *(t* = −4.352 and −4.664, *p* < 0.001 for both, respectively; Figure 2d). This pattern held in both the three-pit and six-pit treatments with no difference *(t* = −0.027, *p* = 0.977).

### 3.2. Second Experiment: Changing Numbers of Pits

**Figure 5:**
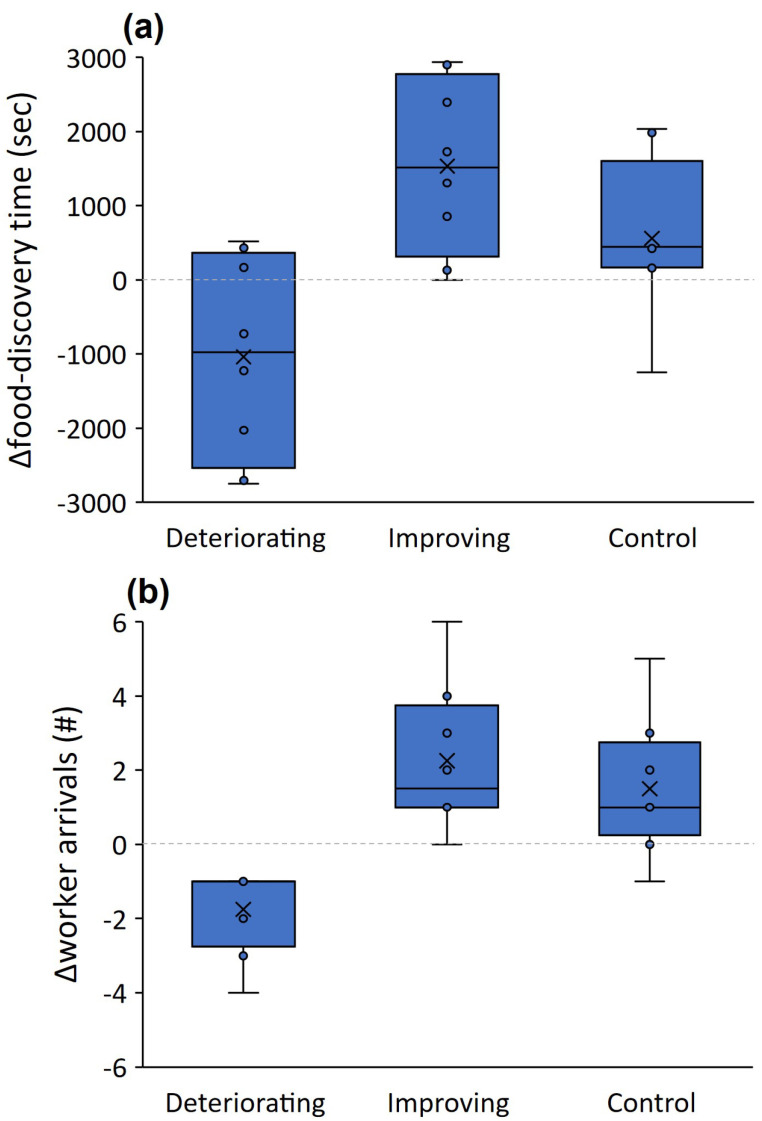
Δfood-discovery time (food-discovery time in the first run minus that in the third run) (**a**) and Δworker arrivals (the number of workers arriving at the food reward in the third run minus that in the first run) (**b**) when conditions deteriorated (pits are deployed only in the third run), when they improved (pits were deployed only in the first and second run), vs. the control (no pits, taken from the first experiment). Lines and × signs in the box centers stand for the median and mean. Boxes and error bars stand for the quartiles and the range.

Δfood-discovery time differed between the treatments of improving and deteriorating conditions *(t* = −4.227, *p* = 0.004) whereas the control was in-between and did not differ from the improving conditions treatment *(t* = −1.231, *p* = 0.258; Figure 5a). This indicates that food was discovered faster in the third run when conditions improved but more slowly when conditions deteriorated. Δworker arrivals differed between improving and deteriorating conditions *(t* = −4.694, *p* = 0.002; Figure 5b) but did not differ from the control *(t* = −0.880, *p* = 0.408). The number of workers falling into pits did not differ between the six-pit and the deteriorating conditions treatments in the third run (U = 22.500, *p* = 0.310). Food-discovery time in the third run did not differ between the six-pit and the deteriorating conditions treatments (U = 28.500, *p* = 0.695). Regarding the comparison between the third run of the control and the improving conditions treatment (both with no pits), food-discovery time and movement speed were similar across treatments (U = 15.500, *p* = 0.083 and U = 29.000, *p* = 0.253, respectively). However, track lengths were around 60% longer in the third run (no pits) of the improving conditions treatment than in the control (U = 5.000, *p* = 0.022; Figure 3a).

## 4. Discussion

Workers of the ant *Cataglyphis niger* discovered the food reward faster and in higher numbers and fell less frequently into pitfall traps in successive runs. We suggest that a process of spatial learning took place here. Track lengths of workers arriving at the food reward were longer and movement was slower in the presence of three pits than in their absence, suggesting a mechanism for the slower food discovery in the presence of pits. Improving and deteriorating conditions (adding or removing pitfall traps) resulted in contrasting results, as expected: food was discovered faster and in higher numbers when conditions improved, and the opposite held true when conditions deteriorated. The control, a constant condition, was in-between. The foraging workers responded rapidly to changing conditions and adjusted their behavior. For instance, improving conditions led to faster food discovery without an impairment imposed by prior conditions and expectations. However, track lengths under improving conditions, when pits were removed after two successive runs, were longer than track lengths in the control (pits were always absent), suggesting that there is still a carryover effect from previous runs. More workers fell into pits further away from the arena entry, as those pits were “shadowed” by pits closer to the arena entry. This pattern may have implications for sit-and-wait predators when choosing their preferred ambush location for prey.

The pitfall traps were costly to the foraging workers in three important ways. First, about a third of workers leaving the nest fell into the traps. Some ant workers pay the ultimate cost and are preyed on whereas others escape (e.g., [27,53]). Even if they escape, the cost is reflected in time, energy loss, or injury. Second, food discovery is delayed, which can be explained by the longer tracks the workers crossed before reaching the food reward, or by their lower movement speed, both demonstrated here. Another untested option is that the most explorative foragers fall into the pits, delaying food discovery. Even when workers do not fall into the pits, the pits still increase the habitat complexity, which is known to delay food discovery or impair foraging success [44,54,55,56]. Such a delay might be costly, if the food is collected by competitors or if the forager is interrupted before locating it and must stop foraging. This leads foraging theory to discount food discovered in delay [57,58]. As the food sources of desert ants are scattered in the habitat and not concentrated in food patches [4,59], the chance of food disappearing owing to late discovery is high.

Workers fell in lower numbers into pits in successive runs. This suggests that workers learn to avoid falling with experience. Learning could be either spatial (avoiding encounters with pits) or associative (associating the pit edge, if it is noticed, with danger and then stepping back). A study on a congeneric ant species demonstrated a similar learning procedure and ant workers fell less frequently to pits with experience and changed their movement paths accordingly [36]. This behavioral change is parallel to learning to overcome obstacles by animals while foraging. Leaf-cutting ants and meat ants, for instance, either learn to travel around an obstacle or cut and clear the way through it [60,61]. Falling in lower numbers into pits during the third run compared to the first run was correlated with faster food discovery and may have been its cause. There was, however, little to no difference in the number of workers falling between the three-pit and six-pit treatments. The reason behind this result could be the ‘zigzag’ arrangement of the three pits so a worker moving in a straight path is likely to encounter a pit at some point, if not the one closest to the arena entrance, then the two downstream. This result emphasizes the importance of “shadow competition”, or the intercept of prey by another predator closer to the arrival source of the prey [62,63,64]. One should thus expect that predators located downstream (more distant from the arena entry) would relocate more often as they encounter prey less frequently. The procedure of pit relocation from less profitable positions, which are “shadowed” by other positions, has been examined in theoretical and experimental studies [62,65,66]. Ambushing predators should perhaps relocate with time anyway, as the ants can learn to avoid them. The suggested increase in the relocation tendency of ambush predators owing to the ant learning to avoid them has never been studied.

The improving or deteriorating conditions in the second experiment led to the expected change: faster food discovery and more workers arriving at the food under improving conditions than deteriorating ones, while the control was in-between these two treatments. The number of workers falling and the food-discovery time were similar in the third run of the six-pit treatment and the third run of the deteriorating conditions treatment, and the food-discovery time was similar in the third run of the control (no pits) and the improving conditions treatment. This suggests that the ant workers rapidly adjust their behavior to changing conditions. The only carryover effect detected was that of the track length of the workers arriving at the food: it was longer in the improving conditions treatment than in the control. The reason could be the mismatch between the memory of the arena with pits in the two previous runs and the current pit-free situation. This increase in track length was not sufficient to translate into delayed arrival at the food. One common cost of learning is using outdated information [67,68], but here, prior information seemed not to interfere strongly with foraging, as it neither delayed arrival at the food nor prevented workers from arriving. Similarly, in a previous experiment with the same species, learning interference (or changing conditions) had little importance for foraging [48]. Future studies should examine whether some workers are more likely to fall into pits than others. For example, it is reasonable to assume a positive correlation between foraging activity and falling likelihood. Thus, although arrival at the food and falling into a pit are mutually exclusive, higher foraging activity might elevate the likelihood of either one taking place. Another possible future direction is to use real predators, such as pit-building antlions, and examine whether the results are similar. Doing so will also reference the other player in the predator–prey interaction [69], and it would be intriguing to examine how the predators respond to the change in the ant worker behavior.

## 5. Conclusions

We demonstrate, here, that desert ant workers learn to avoid pitfall traps and discover food faster with successive runs. If falling into such pits is not always lethal, learning how to avoid pits is adaptive. Although experience played a role, it had little effect on the same foraging traits when conditions improved or deteriorated, i.e., the carryover effects were limited to track length but are not expressed in differences in food-discovery times or the number of workers arriving at the food. Our study demonstrates the effect of habitat complexity on foraging behavior and the improvement of foraging with experience.

## Data Availability

The dataset is attached as a Supplementary Materials Excel file.

## Supplementary Materials

The following supporting information can be downloaded at: https://www.mdpi.com/article/10.3390/biology11060897/s1, Excel File S1: The dataset.
